# Chemotherapy Induced Corneal Changes Assessed by Corneal Confocal Microscopy: A Review

**DOI:** 10.3390/diagnostics14212399

**Published:** 2024-10-28

**Authors:** Eleonora Cosmo, Giulia Midena, Raffaele Parrozzani, Edoardo Midena

**Affiliations:** 1 Department of Neuroscience—Ophthalmology, University of Padova, 35128 Padova, Italy; eleonora.cosmo@unipd.it (E.C.); raffaele.parrozzani@unipd.it (R.P.); 2 IRCCS—Fondazione Bietti, 00198 Rome, Italy; giulia.midena@fondazionebietti.it

**Keywords:** cornea, chemotherapy, corneal confocal microscopy, corneal toxicity, peripheral neuropathy, corneal epithelium

## Abstract

The eye, and the cornea in particular, is a common site of chemotherapy induced toxicity, and ocular side effects of both traditional and novel agents have been reported. Corneal confocal microscopy (CCM) is an in vivo technique that allows for the study of all the corneal layers in an easy, non-invasive and reproducible way via the direct visualization of corneal cell morphologies as well as of sub-basal nerve plexus. Thus, it represents a useful way to identify and monitor chemotherapy induced corneal alterations. This work aims to review the use of CCM in identifying corneal toxicity secondary to chemotherapy treatment, as regards both corneal nerves alterations in the setting of chemotherapy induced peripheral neuropathy (CIPN) and other corneal structure changes, particularly involving the corneal epithelium.

## 1. Introduction

In the few last decades, several chemotherapy drugs have been developed with the aim of treating solid and hematologic tumors. Cytostatic drugs reduce the risk of cancer recurrence through interfering with cell proliferation by targeting cellular DNA or RNA and their metabolism, but they also exert off target effects and kill nontargeted cells with various degrees of toxicity, potentially affecting all organs of the body. Consequently, efforts have been made to develop drugs with lower adverse effects, including immune modulators, checkpoint inhibitors, small molecule inhibitors and monoclonal antibodies possibly conjugated to a cytotoxic drug [1,2]. The eye is a common site of adverse effects from chemotherapy, and ocular side effects of both traditional and novel agents have been reported, ranging from minor to vision-threatening events and affecting also the cornea [3].

The human cornea is an avascular transparent tissue consisting of five layers, which are, from the most superficial to the deepest: the epithelium, a stratified nonkeratinizing squamous layer made up of four to six cell layers which routinely undergo involution, apoptosis and desquamation; the Bowman membrane, an acellular condensate of the most anterior portion of the stroma; the stroma, composed of keratocytes, collagen fibers and extracellular matrix and comprising approximately 80% of the corneal thickness; the Descemet membrane, an amorphous structure; and the endothelium, a monolayer appearing as a honeycomb-like mosaic of hexagonal cells [4]. Furthermore, the cornea is one of the most heavily innervated tissues in the human body, with corneal nerves arising from the ophthalmic branch of the trigeminal nerve, entering the middle stroma and then perforating anteriorly the Bowman membrane, forming the sub-basal nerve plexus, a network of fibers running parallel to the cornea surface [5]. Representing part of the peripheral nervous system, corneal sub-basal plexus may be involved in several peripheral neuropathies, including chemotherapy-induced peripheral neuropathy (CIPN). The assessment of which could be of help in the diagnosis, monitoring and management of the disease.

Corneal confocal microscopy (CCM) is an in vivo technique that allows for the study of all corneal layers in an easy, non-invasive and reproducible way, enabling direct visualization of corneal cell morphologies as well as of sub-basal nerve plexus [6]. Our group previously demonstrated the usefulness of CCM as a biomarker not only for diabetic neuropathy, but also for corneal morphological alterations in post-refractive surgery [7], topical chemotherapy in ocular surface squamous neoplasia [8], vernal keratoconjunctivitis [9], Wilson disease [10] and, more recently, in patients recovered from COVID-19 [11].

The aim of this review is to evaluate the capability of CCM in identifying corneal changes induced by chemotherapy.

## 2. Materials and Methods

The research for potentially relevant articles in the medical literature was conducted via MED-LINE from inception to March 2024 using the following search terms (queried both individually and in combination for advanced research): corneal confocal microscopy, chemotherapy, antineoplastic drugs and adverse effect. Only articles written in English were considered. Inclusion criteria were set to include journal articles and reports that provided relevant insights into the use of CCM as a tool to assess corneal changes induced by chemotherapy. Additional articles were identified by reviewing the references of examined publications. Articles included in the reference list were fully examined by the authors.

After reviewing all the relevant literature, we choose to separately consider the role of CCM in diagnosing and monitoring CIPN, and the utility of the technique in assessing other chemotherapy induced damage on corneal structure aside from the sub-basal nerve plexus, in particular the corneal epithelium.

## 3. Results

### 3.1. CCM in Chemotherapy Induced Polyneuropathy

Due to their high energy activity, fibers of the peripheral nervous system are particularly vulnerable to chemotoxicity [12,13]. Specific groups of chemotherapy drugs, through different mechanisms, are known to induce a peripheral nerve damage, commonly termed CIPN [14,15]. Such a condition, which currently has no approved treatment, represents one of the most common causes of chemotherapy dose reduction and discontinuation, leading to a decrease in the effectiveness of cancer treatment [16]. Prevalence of CIPN ranges approximately from 20 to 85%, depending on the type of neurotoxic drug considered [17]. It is a predominantly sensory neuropathy, characterized by symmetrical and distal involvement, leading to symptoms such as numbness, tingling or abnormal temperature sensations in the distal extremities [14]. Several techniques capable of detecting CIPN are subjective and cannot identify the disease until significant symptomatic changes have developed; on the other side, tests to assess small fiber nerve function are invasive and/or time-consuming [18]. In this context, CCM represents a useful tool to detect in a reproducible and non-invasive way early changes in the corneal sub-basal nerve plexus, and thus to establish the presence of peripheral neuropathy also in chemotherapy treated patients [14]. Studies reporting corneal nerve fibers alterations assessed by means of CCM in chemotherapy treated patients are discussed in the next sections and reported in Table 1.

#### 3.1.1. Platinum Compounds and Taxanes

**Figure 1:**
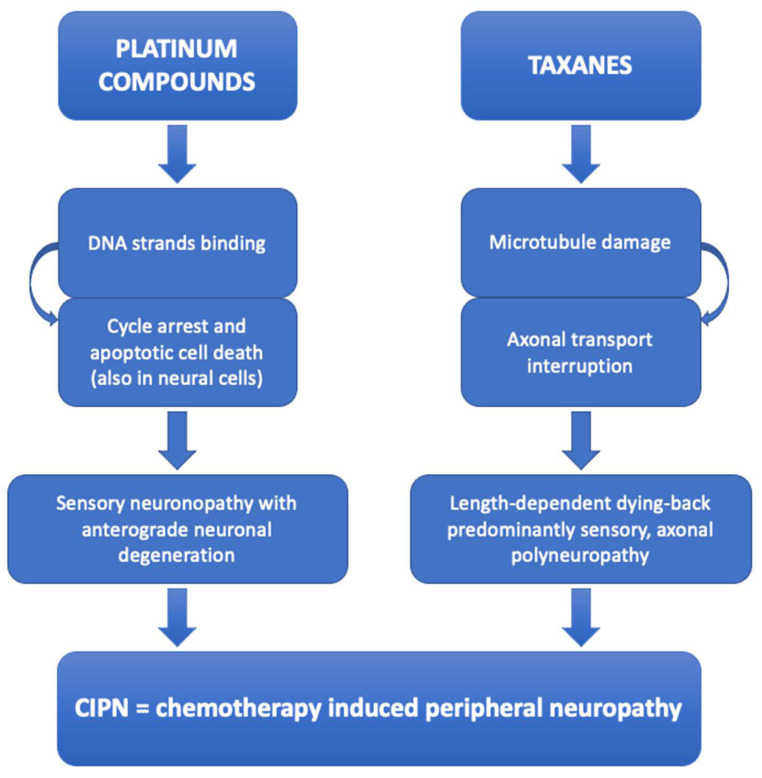
Schematic summary explaining the pathogenic mechanisms of platinum compounds and taxanes induced peripheral neuropathy, which manifests itself even in the corneal sub-basal nerve plexus, thus being visible by means of corneal confocal microscopy.

The two drugs that most frequently cause CIPN are platinum compounds and taxanes—particularly oxaliplatin and paclitaxel, respectively [22]. Indeed, approximately 70 to 85% of patients undergoing such treatments report neuropathic symptoms [29,30]. The former, widely used in the treatment of upper gastrointestinal and colorectal neoplasia, exert their anticancer effect by binding DNA strands and determining cycle arrest and apoptotic cell death, including dorsal root ganglia cells that results in sensory neuronopathy with anterograde neuronal degeneration [31]. The latter, mainly used in the treatment of breast, gynecological and lung cancers, induce microtubule damage and thus affect the axonal transport, leading to a length-dependent dying-back predominantly sensory, axonal polyneuropathy [32] (Figure 1).

**Figure 2:**
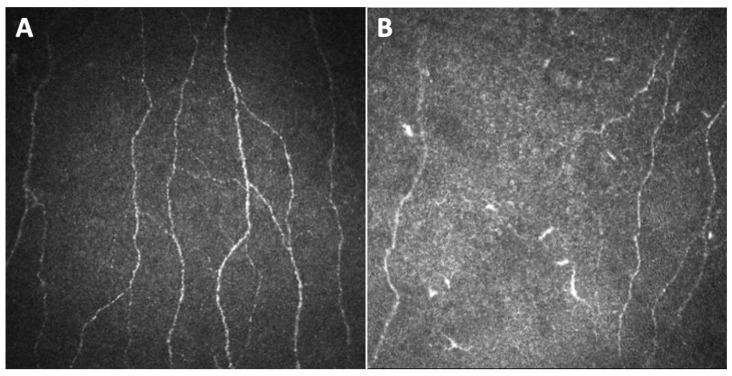
Images (400 × 400 microns) of the sub-basal nerve plexus of an healthy patient (**A**) and of a subject undergoing therapy with oxaliplatin (**B**), captured by means of corneal confocal microscopy (Heidelberg Retina Tomograph III Rostock Corneal Module, Heidelberg, Germany). The plexus of the patient in treatment with oxaliplatin is characterized by a reduced number of thinner nerve fibers compared to that of the healthy subject. Furthermore, there is evidence of immature dendritic cells among the nerve fibers of the patient undergoing chemotherapy treatment.

Campagnolo et al. first shared their experience with CCM as a possible tool to investigate oxaliplatin induced peripheral neuropathy [19]. They studied 15 patients undergoing oxaliplatin therapy for colorectal cancer, finding a significant reduction in the number of fibers and the length density of corneal sub-basal nerve plexus after four cycles of therapy, which persisted after eight cycles. Moreover, in some cases CCM revealed corneal nerves changes before the clinical manifestation of neuropathy. Thus, CCM was demonstrated to be useful in the early diagnosis of oxaliplatin induced peripheral neuropathy, identifying changes in corneal patterns that may also be predictive of coasting effect (Figure 2).

Conversely, a study from Ferdousi et al. on patients affected by upper gastrointestinal cancer treated with oxaliplatin found that corneal nerves fiber length (CNFL) significantly increased after chemotherapy, consistently with nerve sprouting [20]. Such a difference in terms of results may be due to the different time point at which the corneal parameters were assessed. Indeed, in the former study, the decrease in fiber density was found after the fourth cycle of chemotherapy, while in the latter they detected an increase of CNFL after the third cycle, attributing this to an attempt of regeneration of fibers, which may represent an initial reaction of the injured sub-basal nerve plexus, followed by a degeneration of the nerves. Except for a work by Bennedsgaard et al., which did not observe a difference in corneal nerves parameters between patients with and without small fiber polyneuropathy 5 years after adjuvant chemotherapy with docetaxel or oxaliplatin [21], almost all the other studies in the literature assessing CIPN as identified by CCM showed changes of the sub-basal nerve plexus. In particular, Chiang et al. extensively studied a population of patients treated for cancer with oxaliplatin or paclitaxel by means of CCM [22,23,33,34]. The sub-basal nerve assessment was performed 3 to 24 months after the end of the treatment, and the tested parameters, obtained through an automated image analysis software (ACCMetrics, The University of Manchester Intellectual Property UMIP, Manchester, United Kingdom), were CNFL, corneal nerve fiber density (CNFD), corneal nerve branch density (CNBD), corneal nerve fiber area (CNFA), inferior whorl length (IWL) and average nerve fiber length (ANFL). They found evidence of sub-basal corneal nerve loss in patients with cancer treated with paclitaxel and oxaliplatin well after treatment interruption, advocating CCM as a useful tool in the monitoring of CIPN. Furthermore, they demonstrated that corneal nerve reduction, particularly in IWL decrease, correlated with worse upper limb function [22], while no association was found between corneal parameters and the Ocular Surface Disease Index (OSDI), used to measure ocular surface discomfort [23], nor with tears fluid’s levels of Substance P, a neuropeptide expressed by sensory nerves, involved in both pain signaling and the regulation of epithelial and neural health [34]. Finally, they also demonstrated by means of CCM an increase, in the oxaliplatin treated group but not in the paclitaxel group, of immature dendritic cells, which are potent antigen-presenting cells [33]. Such an increase, associated with higher drug cumulative dose and treatment cycles, may be indicative of a sustained, chronic low-level inflammation, reflecting a neuroimmune interaction between these immune cells and corneal nerves (Figure 2).

Other studies have focused on the role of neuroinflammation as a mechanism of damage in CIPN and thus on the search, among the corneal nerve fibers, for dendritic cells [35,36]. The presence of dendritic cells by means of direct visualization via CCM has been explored in a patient affected by breast cancer treated with paclitaxel and trastuzumab, followed for 58 weeks after the baseline examination. The same identical area of the sub-basal nerve plexus was captured during follow-up, and the plexus morphology did not show modification. Since this patient has never developed CIPN, the finding is consistent with previous studies demonstrating that corneal nerve dysfunction is more evident in patients with CIPN, supporting the usefulness of CCM for monitoring nerve function in patients receiving paclitaxel. However, a local burst of dendritic cells at 6 and 11 weeks after baseline examination was detected. The authors hypothesized this may indicate ongoing inflammation or immune-mediated reaction most likely determined by paclitaxel rather than by trastuzumab, since after paclitaxel discontinuation due to side effects a return to normal levels of dendritic cells was documented. They concluded that longitudinal in vivo CCM could be helpful to generate a comprehensive clinical picture, representing, with corneal structures acting as biomarker, a diagnostic tool for the assessment of adverse events during paclitaxel therapy.

Finally, two other studies analyzed corneal nerves changes by CCM in CIPN, in patients receiving oxaliplatin for gastrointestinal cancer [24] and in patients with different types of cancer exposed to a regimen of neurotoxic chemotherapeutic agents including, besides paclitaxel and platinum compounds, also Bortezomib-Thalidomide-Dexamethasone (VTD), or Cyclophosphamide-combined treatments [25]. The former showed that corneal nerve density did not present significant changes after chemotherapy; however, it was modestly correlated with clinical peripheral neuropathy after 20 weeks of chemotherapy, when peripheral neuropathy usually becomes severe. The authors concluded that corneal nerve density could not be considered a reliable surrogate biomarker for oxaliplatin neuropathy. Nevertheless, the same authors highlighted that patient attrition due to morbidity and mortality could potentially have biased the results [24]. The work of Riva et al. found that CNFL was not significantly reduced after chemotherapy; however, corneal nerve fiber density/tortuosity ratio significantly decreased after therapy, demonstrating correlation with the number of chemotherapy cycles. These authors suggested that CCM could represent a helpful, non-invasive tool which shows promise for the diagnosis of CIPN.

#### 3.1.2. Other Chemotherapy Drugs

Even if the most common antineoplastic agents showing neurotoxicity are platinum compounds and taxanes, several other drugs have been studied as regards the damage induced to the peripheral nervous system, and in particular to corneal nerves, identified by CCM.

Ferrari et al. reported a case treated with capecitabine, an antineoplastic drug known to induce peripheral sensory neuropathy [37], for a metastatic colorectal carcinoma, showing on CCM significant anomalies in morphology and number of corneal nerves in the sub-basal epithelial plexus and the stroma [26]. In particular, they found increased beading, tortuosity, sprouting and fading extremities of the corneal nerves.

Bortezomib, a proteasome inhibitor used in the treatment of multiple myeloma, has also been studied as a chemotherapy agent responsible for neurotoxicity and corneal changes visible on CCM images. As regards the mechanism that underlies peripheral nerve damages, bortezomib reduces NF-κB activity, which is involved in DNA transcription and cytokine production required for cellular growth, and mitochondrial and endoplasmic reticulum damage has been demonstrated in Aδ and C fiber axons, Schwann cells and the dorsal root ganglia. In addition, it also induces aerobic glycolysis leading to an increase in metabolites sensitizing sensory neurons to pain [15]. A study by Cocito et al. reported an altered CCM neural pattern in patients affected by multiple myeloma treated with bortezomib, compared to healthy subjects [27]. Thus, the authors suggested a possible role for CCM as a non-invasive tool for early detection of bortezomib induced neural damage, predictive for the development of clinically significant peripheral neuropathy.

Finally, bortezomib in combination with thalidomide and dexamethasone, together with other drugs such as cyclophosphamide-combined treatments, besides paclitaxel and platinum compounds, were tested in a study by Riva et al. as regards their association with corneal nerve density and morphology assessed by CCM, to determine if this technique could be useful in monitoring the neurotoxic effects of chemotherapy compared to epidermal nerve quantification [25]. As previously stated, nerve fiber density/tortuosity ratios showed a significant decrease after therapy, suggesting that CCM could represent a promising mean for the diagnosis of CIPN.

Also biological drugs inhibiting the epidermal growth factor receptor (EGFR), including erlotinib, cetuximab, panitumumab and depatuxizumab mafodotin, have been associated with changes of the sub-basal nerve plexus identified by CCM, together with other alterations of the corneal epithelium that will be largely discussed in the following chapter [28,38,39].

### 3.2. CCM in Other Chemotherapy Induced Damage on Corneal Structure

Corneal toxicity due to chemotherapy drugs does not involve only the sub-basal nerve plexus as a manifestation of CIPN; it also affects other corneal structures. CCM, being able to visualize all corneal layers by providing images with a very high resolution and magnification, may represent a tool capable of diagnosing and monitoring all possible corneal side effects of antineoplastic, thus supporting physicians to assess toxicity grade and guiding them to potential changes in therapeutic regimen. CCM findings of corneal damage induced by chemotherapy on structures other than sub-basal nerve plexus are reported in Table 2, and hereafter discussed.

The first chemotherapy induced corneal alterations assessed by means of CCM were reported in a 69-year-old female patient on a low dose of tamoxifen since two years for breast cancer [40]. Tamoxifen is an estrogen antagonist with well-documented ocular side effects [49]. The authors described the presence of multiple tiny crystalline deposits in the deeper stroma of both eyes in an asymptomatic patient with unremarkable anterior segment findings upon slit lamp examination. Thus, they concluded that CCM could be a useful adjunct in monitoring tamoxifen crystalline keratopathy by helping with the detection of subclinical deposits that may be invisible on slit lamp examination.

Corneal toxicity induced by trastuzumab emtansine has also been assessed by means of CCM. Trastuzumab emtansine is a biological drug used in the treatment of human epidermal growth factor receptor-2 (HER2) positive metastasized breast cancer, formed by a monoclonal anti-HER2 antibody, trastuzumab, conjugated with a cytotoxin, emtansine, which is a tubulin-inhibitor. The monoclonal anti-HER2 antibody targets tumor cells, afterwards the conjugate is internalized and, within the cells, it undergoes lysosomal degradation, releasing the cytotoxin [50]. Such a mechanism allows for the targeting of cancer-specific antigens, theoretically limiting systemic adverse effects. However, HER2 is expressed, albeit at low levels, by the epithelial cells of most healthy tissues, including in particular and with a stronger expression in the ocular surface epithelium [51]. Indeed, drugs inhibiting epidermal growth factor receptor have been linked with corneal epithelial lesions from punctate keratopathy to corneal melting [41]. A study by Kreps et al. reported the case of a patient under treatment with trastuzumab emtansine for metastasized breast cancer, complaining of blurred vision, with multiple intraepithelial spherical lesions in the corneal midperiphery, correspondent, at CCM examination, to multiple hyperreflective lesions in the basal epithelium associated with pleiomorphic epithelial cells [41]. A diagnosis of trastuzumab emtansine—associated epitheliotoxicity—was made, and corneal findings remained stationary after treatment with autologous serum drops. Another cross-sectional, prospected study conducted on 12 patients undergoing therapy with trastuzumab emtansine demonstrated the presence of coarse cystoid lesions in the deep corneal epithelial cells, mainly in the midperipheral cornea. Such changes were absent in two patients who stopped treatment, suggesting a complete reversibility of the chemotherapy induced keratopathy [42].

Other biological drugs, whose mechanism is the inhibition of EGFR, have been studied as regards corneal alterations, visible by means of CCM. Two cases of cornea verticillata after treatment with vandetanib (i.e., a dual anti-EGFR and vascular endothelial growth factor receptor 2 (VEGFR2) tyrosine kinase inhibitor) for medullary thyroid carcinoma were reported [43]. CCM images showed hyperreflective deposits, smaller than the affected cells, in the corneal epithelium and subepithelial nerve plexus, as well as bright microdots throughout the stroma. The authors speculated that such deposits could represent drug–lipid complexes, or a secondary effect of EGFR inhibition consisting in impairment in the normal epithelial turnover and cell migration. A prospective study demonstrated, in a group of patients under treatment with EGFR inhibitors (including erlotinib, cetuximab and panitumumab), worsening, not only of dry eye signs, but also of epithelial cells density and sub-basal nerve plexus density, as evaluated by CCM [38]. EGFR is overexpressed in several tumors, indeed molecular targeted therapy for cancer using EGFR inhibitors is showing good results and growing evolution. However, since EGFR also stimulates corneal epithelial cells proliferation via lacrimal gland secretion, its inhibition affects corneal epithelium with consequent keratopathy, which may be manifest or identifiable by CCM.

**Figure 3:**
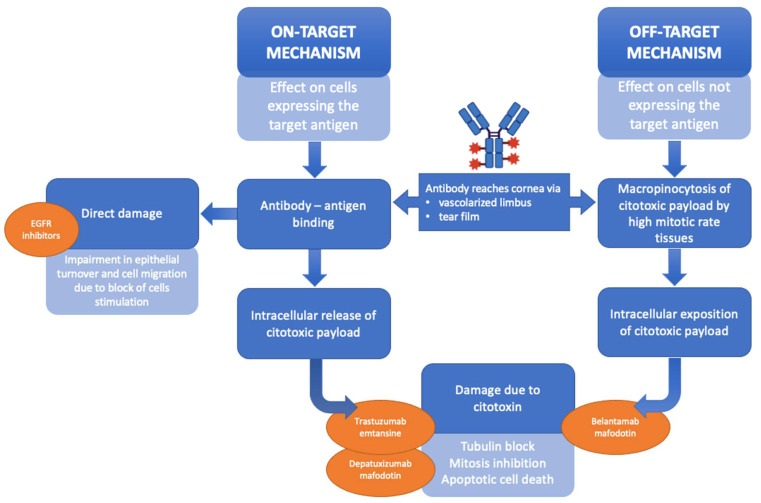
Schematic summary explaining the on-target and off-target mechanisms exerted by different chemotherapy drugs consisting in antibodies with or without conjugated citotoxin, causing corneal side effects. Epidermal growth factor receptor (EGFR) inhibitors (such as vandetanib, erlotinib, cetuximab and panitumumab) cause direct damage to epithelial cells by inhibiting EGFR with consequent impairment in the normal epithelial turnover and cell migration. Antibody-drug-conjugated (ADC), such as trastuzumab emtansine and depatuxizumab mafodotin, lead to corneal injury mainly via on-target mechanisms, since the antigens target of these ADC (HER-2 for trastuzumab and EGFR for depatuxizumab) are expressed in the corneal epithelium. Whereas, belantamab mafodotin causes corneal changes primarily with off-target mechanisms, since belantamab target (i.e., B-cell maturation antigen) is not expressed in the cornea.

Parrozzani et al. were the first to prospectively analyze, using CCM, corneal side effects induced by depatuxizumab mafodotin (ABT-414), an EGFR inhibitor, in a group of patients affected by EGFR-amplified recurrent glioblastoma [28,39]. CCM images showed multiple and diffuse hyperreflective white round spots and round cystic structures in the corneal basal epithelium, as well as a progressive fragmentation followed by full disappearance of the sub-basal nerve plexus fibers. Furthermore, the authors demonstrated that such changes were completely reversible and that resolution of signs and symptoms ranges between 24 and 53 days after the end of treatment. In this biological drug, the monoclonal antibody that binds EGFR (depatuxizumab) is conjugated with monomethyl-auristatin-F, an antimitotic agent that inhibits cell division by blocking the polymerization of tubulin. Thus, the mechanism inducing corneal toxicity may be due to the cytotoxic activity directly caused by the drug on the corneal epithelium representing the target [52]. A second hypothesis regards the so-called off-target mechanism (uptake of the unconjugated cytotoxin by tissue having a specific tropism for it), which means that the drug may undergo intracellular uptake via endocytosis in those cells characterized by high replication and differentiation rate, such as corneal limbal stem cells (Figure 3) [50].

**Figure 4:**
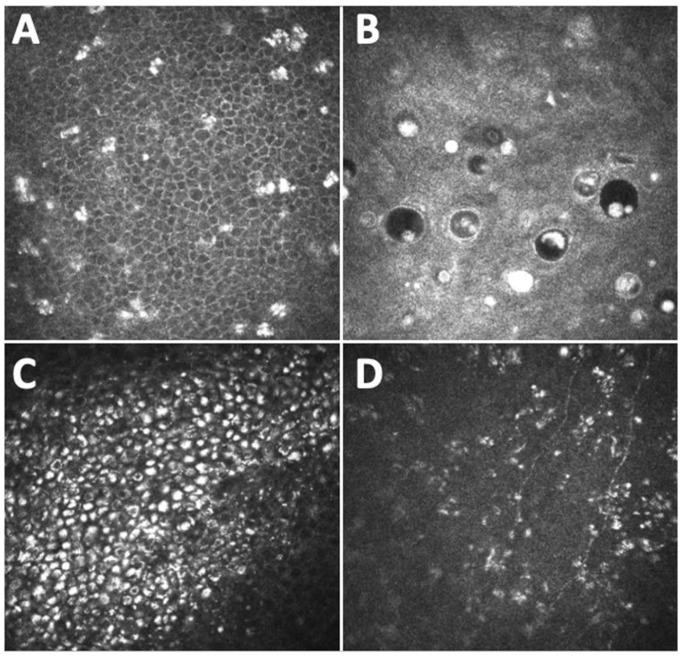
Corneal confocal microscopy images (400 × 400 microns) captured with Heidelberg Retina Tomograph III Rostock Corneal Module (Heidelberg, Germany) of patients undergoing therapy with belantamab mafodotin (belamaf), showing corneal microcyst-like epithelial changes induced by the therapy. Specifically, image in (**A**) shows hyperreflective opacities within the corneal basal epithelium; image in (**B**) demonstrates the presence of intraepithelial round microcystic structures consisting of a hyperreflective wall; image in (**C**) shows a cluster of epithelial hyperreflective deposits, which are also evident in the sub-basal nerve plexus layer (as evident in image in (**D**)), characterized by reduced and thinner fibers.

Recently, considerable attention has been paid to belantamab mafodotin (belamaf) and its corneal side effects. Belamaf is a novel antibody drug conjugate developed for the treatment of relapsing remitting multiple myeloma, composed of an humanized immunoglobulin targeting B-cell maturation antigen (BCMA) conjugated to a cytotoxic payload, the microtubule-disrupting agent known as monomethyl auristatin F [53]. CCM has been useful to assess, initially during trial phases and then also in studies from real life settings, corneal microcyst-like epithelial changes induced by the therapy (Figure 4). Images captured with CCM in patients included in the DREAMM-2 study showed hyperreflective opacities within the corneal epithelium, most prominent in the wing and basal cells rather than in the superficial cells [44]. Other reports have described, in patients treated with belamaf, hyperreflective deposits in the basal epithelium and the sub-basal nerve plexus layer, diffusely over the entire corneal surface, arranged in clusters, and small degenerative intraepithelial microcysts consisting of a hyperreflective wall at a more superficial level [45,46].

Such changes have been demonstrated to be reversible after treatment interruption, and CCM represents a tool to implement the current clinical scale used for grading belamaf induced corneal toxicity, and to help clinicians find the correct dose for each patient, rather than stopping the treatment due to severe ocular adverse effects. There are several hypotheses about the mechanisms by which belamaf induces corneal epithelial damage, including both the on-target and the off-target scheme, as was previously cited in relation to other antibodies conjugated drugs. However, given that the belantamab target (i.e., BCMA) is not expressed in the cornea, the belamaf induced microcyst-like epithelial changes may primarily represent an off-target mechanism [44]. It has been suggested that belamaf, after reaching the corneal epithelium, either through the vascularized area of the limbus or through the tear film after secretion from the lacrimal gland, may be internalized by progenitor cells via micropinocytosis, thereby initiating apoptosis. Therefore, the hyperreflective opacities visualized at CCM may represent the accumulation of apoptotic end products in the intercellular spaces of corneal epithelium [44,46].

A few other reports have described corneal epithelium alterations as seen by CCM in patients treated with different chemotherapy drugs, such as capecitabine and lapatinib and high-dose cytarabine [47,48]. Thus, CCM is confirmed to be a useful technique for diagnosing and monitoring chemotherapy induced corneal epithelium damage.

### 3.3. Limits and Future Directions

This work reviewed how CCM has been demonstrated to be a tool capable of identifying chemotherapy induced corneal damage, both as regards sub-basal nerve plexus changes in the context of CIPN and epithelial and/or stromal alterations. However, the use of such a technique is still mainly confined to scientific research, albeit with some limits, whereas in clinical practice this tool is not widely employed. The limitations are primarily due to the difficulty of the image analysis. First of all, there is a lack of standard criteria regarding the number of images to analyze as well as the methods used to detect cells and especially nerve fibers (as Table 1 highlights). Originally, manual analysis was used, but it was a tedious, subjective and time-consuming procedure requiring considerable expertise. Subsequently, an automatic or semi-automatic approach was introduced [54,55], and recently a number of fully automated deep learning methods based on convolutional neural networks have been developed to analyze corneal nerve parameters [56,57,58,59], but differences among groups and studies are still huge and standardization for CCM images analysis is still missing. Thus, for the future, the search for uniformity not only in the acquisition but also in the analysis of CCM images is desirable, with the aim of spreading the use of this technique also among clinicians to help them in diagnosing and monitoring chemotherapy induced corneal damage. The introduction of artificial intelligence in the evaluation of CCM data may help with this objective.

## 4. Conclusions

Chemotherapy induced corneal damages are multiple. Antineoplastic drugs, in particular platinum compounds and taxanes, may induce peripheral neuropathy. The corneal sub-basal nerve plexus, part of the peripheral nervous system, may be involved in CIPN, and corneal nerve damage could be easily identified by means of CCM, which represents a non-invasive technique to diagnose and monitor the disease. At the same time, CCM has been revealed to be a useful tool for identifying other corneal alterations secondary to chemotherapy, in particular microcystic-like epithelial changes induced by biological drugs and antibody drug conjugates. Once again, CCM may support clinicians not only in assessing and better characterizing such kinds of chemotherapy induced corneal toxicity, but also in adjusting the drug dosage in order to reduce adverse effects.

## Figures and Tables

**Table 1:** Studies reporting corneal nerve fibers alterations assessed by means of CCM in chemotherapy treated patients.

Study	Chemotherapy Agent	Design, no. of Patients	Nerve Parameters	Significant Results	Methods	Clinical Relevance
Campagnolo et al., 2013 [19]	Oxaliplatin	Longitudinal, 15	Length density	→ reduction	n.s.	Prediction of coasting effect
			Number of fibers	→ reduction		
			Number of beading	/		
			Tortuosity	/		
Ferdousi et al., 2015 [20]	Oxaliplatin, cisplatin	Cross-sectional, 21 Longitudinal, 13	CNFD	→ reduction vs. controls	Analysis of 6 images (3 from each eye) with CCMetrics *	Aid in diagnosis of CIPN
			CNBD	→ reduction vs. controls		
			CNFL	→ reduction vs. controls, increase in follow-up		
Bennedsgaard et al., 2020 [21]	Oxaliplatin, docetaxel	Cross-sectional, 63	CNFL	/	Fiber counted automatically	/
			CNFD	/		
			CNBD	/		
Chiang et al., 2021 [22]	Oxaliplatin, paclitaxel	Cross-sectional, 70	CNFL	→ reduction	Analysis of 8 images from the central cornea and 3 to 5 images from the inferior whorl region with ACCMetrics #	Aid in the monitoring of nerve function in patients undergoing chemotherapy
			IWL	→ reduction		
			ANFL	→ reduction		
			CNFD	→ reduction		
			CNFA	→ reduction		
			CNBD	/		
Chiang et al., 2021 [23]	Paclitaxel	Cross-sectional, 29	CNFL	→ reduction	Analysis of 8 images from the central cornea and 5 images from the inferior whorl region with ACCMetrics #	/
			IWL	→ reduction		
			CNFD	→ reduction		
			CNBD	/		
Tyler et al., 2022 [24]	Oxaliplatin	Cross-sectional, 23	Corneal nerve density	→ correlation with clinical peripheral neuropathy	Semi-automated analysis with software ImageJ—NeuronJ §; Automated analysis with customized deep learning-based approach deepNerve	Aid in diagnosis of CIPN
			Corneal nerve lenght	/		
Riva et al., 2022 [25]	Platinum compounds, paclitaxel, bortezomib-thalidomide-dexamethasone, cyclophosphamide-combined treatments	Longitudinal, 73	CNFL	/	Deep learning technique Convolutional Neural Network	Aid in diagnosis of CIPN
			Tortuosity	/		
			CNFL/tortuosity	→ reduction		
Ferrari et al., 2010 [26]	Capecitabine	Case report, 1	Beading, tortuosity and sprouting	→ increase vs. age-matched control	n.s.	Aid in diagnosis and follow-up of CIPN
Cocito et al., 2015 [27]	Bortezomib	Cross-sectional, 26	Nerve fiber length	→ reduction	n.s	Prediction for clinically significant peripheral neuropathy
			Nerve fiber number	→ reduction		
			Beadings number	→ reduction		
			Nerve fiber tortuosity	→ increase		
Parrozzani et al., 2020 [28]	EGFRi (depatuxizumab mafodotin)	Longitudinal, 15	Fragmentation/disappearance of fibers	→ present in all patients	n.s.	Ocular side effects due to ABT-414 can be manageable

no.: number; n.s.: not specified; CNFD: corneal nerve fiber density; CNBD: corneal nerve branch density; CNFL: corneal nerve fiber length; IWL: inferior whorl length; ANFL: average nerve fiber length; CNFA: corneal nerve fiber area; EGFRi: epidermal growth factor receptor inhibitor; CIPN: chemotherapy indiced peripheral neuropathy. * MA Dabbah; Imaging Science and Biomedical Engineering, University of Manchester, Manchester, UK. # The University of Manchester Intellectual Property UMIP, Manchester, UK. § National Institutes of Health, Bethesda, Rockville, MD, USA.

**Table 2:** CCM findings of corneal damage induced by chemotherapy on structures other than sub-basal nerve plexus.

Chemotherapy Agent	Study	CCM Findings	Corneal Layer Affected	Clinical Relevance
Tamoxifen	Tarafdar et al., 2012 [40]	Multiple tiny crystalline deposits	Stroma	Aid in monitoring tamoxifen crystalline keratopathy
Trastuzumab emtansine	Kreps et al., 2018 [41]	Multiple hyperreflective lesions associated with pleiomorphic cells	Basal epithelium	/
	Deklerck et al., 2019 [42]	Coarse cystoid lesions	Deep epithelium	Indication for adjusting systemic treatment
EGFRi				
Vandetanib	Arriola-Villalobos et al., 2018 [43]	Hyperreflective deposits	Epithelium and subepithelial nerve plexus	/
		Bright microdots	Stroma	
Depatuxizumab mafodotin	Parrozzani et al., 2020 [28]	Multiple and diffuse hyperreflective white round spots and round cystic structures	Basal epithelium	Indication for adjusting systemic treatment
Belantamab mafodotin	Farooq et al., 2020 (DREAMM-2 Study) [44]	Hyperreflective opacities	Epithelium (mainly basal)	Indication for adjusting systemic treatment
	Marquant et al., 2021 [45]	Clusters of hyperreflective material and small degenerative intraepithelial microcysts, mainly consisting of a hyper-reflective wall	Basal epithelium and sub-basal nerve plexus	
	Mencucci et al., 2022 [46]	Hyperreflective opacities	Epithelium	
Capecitabine and lapatinib	Di Staso et al., 2021 [47]	Irregular cellular population and a mosaic pattern consisting mostly of hypo-reflective cells and cystic changes	Epithelium	Indication on adequate therapy for ocular side effects of capecitabine
Cytarabine	Özcan et al., 2021 [48]	Highly reflective disseminated granular and irregular opacities	Basal epithelium	Indication on adequate therapy for ocular side effects of cytarabine

EGFRi: epithelial growth factor receptor inhibitors.

## Data Availability

The data presented in this study are available in the article. Eventually, additional data will be available on request from the corresponding author.
